# Health Status, Life Habits, and Social Background among the JPHC Study Participants at Baseline Survey

**DOI:** 10.2188/jea.11.6sup_57

**Published:** 2007-11-30

**Authors:** Masamitsu Konishi, Hirokazu Kondou, Katsutoshi Okada

**Affiliations:** 1 Department of Preventive Cardiology, National Cardiovascular Center.; 2 Department of Public Health, Ehime University School of Medicine.

**Keywords:** health status, past history, medical treatment, life habits, occupation, social background

## Abstract

A self-reported questionnaire on the health status, life habits, and social background was conducted at baseline in the Japan Public Health Center-based Prospective Study on Cancer and Cardiovascular Diseases (JPHC study). This report presents the outcome of the study regarding past or family history of various diseases, medical treatment, life habits such as physical labor or sports, and social background among study participants. In both cohorts I and II, prevalent past and family history included hypertension, stroke, and cancer, whereas the prevalence of coronary heart diseases was historically low. The prevalence of a past history of hypertension and stroke was higher in the northern part of Japan, Ninohe, and Yokote, and lower in Okinawa, compared to the other districts. The prevalence of participants with a history of stomach cancer and liver cancer was higher in Arikawa than in other districts. The frequency of participants who took medication from doctors ranged from 20% to 30%, higher in the Tohoku areas, and lower in Okinawa compared to the other districts. All districts showed a high rate of over 70% for the participation rate for basic health examination conducted by the local government, The rate was particularly high in the Tohoku area where a high prevalence of a history of hypertension was found. The frequency of persons who had a chance to participate in sports or physical exercise was high in Okinawa and Suita subcohort 2, although the mean total physical activity (both at work and for leisure time) was lowest in the latter subcohort. No substantial differences were found in compositions of personality among districts. The frequency of more active and positive persons, however, was relatively higher in urban areas and lower in Okinawa compared to the other districts. The association between the differences of health status, life habits, and social background and the occurrence of various lifestyle-related diseases will be clarified in a follow-up study within the JPHC study.

## INTRODUCTION

Many epidemiologic and pathologic studies indicate that etiology of cardiovascular disease (CVD) varies among geographically and occupationally different groups^1^^-^^4^^)^.Compared with the pattern of the CVD in the United States and Europe, the incidence rates in Japan are higher for stroke and lower for ischemic heart disease. However, this special pattern of CVD is not evenly distributed throughout Japan. The incidence rate of stroke is higher in the northern part than in the western part of Japan, and the incidence rate of ischemic heart disease is higher in urban populations than in rural populations.

For urban populations, especially white collar workers, the main risk factors for CVD, such as stroke and myocardial infarction, are hypertension, hyperlipidemia, smoking, and abnormal glucose metabolism, which are compatible with those reported from the United States and Europe^5^^, ^^6^^)^. On the other hand, serum total cholesterol is unlikely to be associated with the incidence of stroke and myocardial infarction, whereas blood pressure is positively associated for rural populations^7^^-^^10^^)^. It is considered that these differences in risk factors for CVD between urban and rural populations are based on environmental factors.

In the JPHC study, we assessed the health status, life habits, and social background of study participants at the baseline survey using a self-reported questionnaire. This report presents the outcome of the study regarding past or family history of various diseases, medical treatment, life habits such as physical labor or sports, and social background of the participants.

## MATERIALS AND METHODS

The baseline survey for cohort I was conducted between 1988 and 1990 in 5 selected districts under the administration of the public health center. A survey for cohort II was conducted in 6 other districts between 1993 and 1995.

Self-reported questionnaires were distributed to all study participants to examine the following characteristics: health status; lifestyles such as smoking, alcohol consumption, and diet; occupation; and social background. The questionnaire used for cohort II was a revised version of the questionnaire used for cohort I. There were some differences in the contents of the questionnaire used in the two cohorts, which made it difficult to compare the some of data of the two cohorts.

In cohort I, the questionnaire inquired about a past history of ischemic heart disease, including angina pectoris and myocardial infarction. In cohort II, only a history of myocardial infarction was included. Because cohort I was focused on detecting cancer risks, a history of hyperlipidemia was included only in cohort II. For family history, ischemic heart disease was part of the cohort I questionnaires, but heart diseases including ischemic heart disease and unknown heart disease were asked about in cohort II. The term “hypercholesterolemia” was used in cohort I questionnaires, but it was changed to “hyperlipidemia” in cohort II questionnaires.

Responses to questions about participation in basal health checkups conducted by the local government required “no” or “yes” answers in both cohorts, but the recognition of results was included only in cohort II. In cohort I, the questionnaire asked participants to choose one of three categories, “decrease,” “no change,” or “increase,” for change in body weight of 5 kg or more compared with weight at 20 years old, In cohort II, respondents were asked directly about their current body weight and at 20 years old.

The opportunities for participating in sports and physical exercise were part of the questionnaires for both cohorts according to one of five categories: “almost never,” “1 to3 days/month,” “1 to 2 days/week,” “3 to 4 days/week,” or “almost every day.” In cohort II, working hours were additionally included. Questions about numbers of siblings and numbers of children were included only in cohort I. A history of education was also only included in cohort I questionnaires.

In cohort I, the baseline survey was conducted for residents aged 40 to 59 years. The Katsushika subcohort conducted the survey for residents aged 40 and 50 years at the time of health screening at the public health center. In cohort II, the baseline survey was conducted for all residents aged 40 to 69 years, except the Suita subcohort 1, in which the survey was conducted for all residents aged 40 and 50 years. We analyzed the results of questionnaires separately among districts in each cohort to compare the results.

## RESULTS

### Health Status

#### 1) Past History

Of all diseases, hypertension was the most prevalent past history in cohort I (Table 1a,b): 11.5% to 17.4% for males and 11.7% to 15.1% for females. The prevalence of hypertension did not differ among districts, except for a relatively higher prevalence in Ninohe and Yokote and lower prevalence in Ishikawa.

**Table 1a.** Prevalence of medical history of chronic disease in cohort I males (%).

	Ninohe	Yokote	Saku	Ishikawa	Katsushika	Total
N	4,228	5,471	5,410	5,156	2,764	23,029
Stroke	1.1	0.6	0.4	0.2	0.4	0.5
Ischemic Heart Disease	1.3	1.2	1.7	1.1	1.6	1.4
Hypertension	17.0	17.4	15.1	11.5	9.7	14.6
Diabetes Mellitus	6.3	5.0	6.5	3.9	3.9	5.2
Chronic Hepatitis or Liver Cirrhosis	2.2	1.7	2.0	1.0	1.7	1.7
Peptic Ulcer	12.0	12.4	15.5	9.1	13.9	12.5
Biliary Stone	2.0	1.4	2.6	4.1	2.0	2.5
Cancer	1.4	1.5	1.6	1.4	0.3	1.3
Stomach	0.5	0.8	0.7	0.4	0.0	0.5
Lung	0.1	0.0	0.1	0.3	0.0	0.1
Colon and Rectum	0.2	0.1	0.3	0.2	0.0	0.2
Liver	0.5	0.4	0.3	0.4	0.2	0.4
Breast	0.1	0.0	0.0	0.0	0.0	0.0
Uterus	-	-	-	-	-	-
Other	0.1	0.2	0.2	0.1	0.1	0.1

**Table 1b.** Prevalence of medical history of chronic disease in cohort I females (%).

	Ninohe	Yokote	Saku	Ishikawa	Katsushika	Total
N	4,873	6,283	5,477	5,297	3,984	25,914
Stroke	0.4	0.3	0.2	0.2	0.2	0.2
Ischemic Heart Disease	1.0	1.2	0.9	0.6	0.9	0.9
Hypertension	15.1	14.6	13.2	11.7	7.8	12.8
Diabetes Mellitus	2.4	2.2	2.8	2.3	1.3	2.2
Chronic Hepatitis or Liver Cirrhosis	0.7	0.6	0.8	0.5	0.7	0.7
Peptic Ulcer	6.4	5.5	8.2	4.5	6.0	6.1
Biliary Stone	2.3	2.1	2.6	4.7	2.0	2.7
Cancer	2.9	2.7	3.4	2.5	1.6	2.7
Stomach	0.3	0.4	0.4	0.2	0.1	0.3
Lung	0.0	0.1	0.1	0.1	0.0	0.1
Colon and Rectum	0.1	0.1	0.2	0.1	0.1	0.1
Liver	0.2	0.1	0.3	0.1	0.1	0.2
Breast	0.5	0.7	0.8	0.3	0.5	0.6
Uterus	1.4	1.0	1.5	1.4	0.7	1.2
Other	0.3	0.3	0.2	0.2	0.2	0.2

A history of stroke in cohort I was 0.2% to 1.1% for males and 0.2% to 0.4% for females. No difference among districts was found except for Ninohe, which showed a relatively high rate in males. A history of myocardial infarction or angina pectoris was 1.0% to 1.7% in males and 0.6% to 1.2% in females. The history of cancer in cohort I was 1.4% to 1.6% in males and 2.5% to 3.4% in females. No significant differences among districts were found in these past histories.

In cohort II, the prevalence of hypertension was 19.4% to 25.1% in males and 15.8% to 21.5% in females (Tables 2a,b). No significant differences among districts were found. The history of stroke was 1.1% to 1.7% for males and 0.3% to 0.9% for females, with no differences among districts. The history of myocardial infarction was 1.0% to 2.6% in males and 0.5% to 0.9% in females, except for Suita subcohort 2, which showed a slightly higher prevalence in males. A history of cancer was reported by 0.9% to 2.7% of the males and 1.6% to 3.2% of the females. Both males and females in Kashiwazaki showed a lower prevalence. The history of stomach cancer and liver cancer was more frequent in Arikawa than other districts.

**Table 2a.** Prevalence of medical history of chronic disease in cohort II males (%).

	Kasama	Kashiwa zaki	Tosa yamada	Arikawa	Miyako	Suita 1	Suita 2	Total
N	9,453	1,520	3,561	4,983	4,779	2,907	2,094	29,297
Stroke	1.1	1.5	1.3	1.4	1.3	0.3	1.7	1.2
Myocardial Infarction	1.6	1.4	1.9	1.7	1.0	0.7	2.6	1.5
Hypertension	19.4	25.1	18.7	19.1	19.6	11.7	21.6	19.0
Diabetes Mellitus	7.9	4.7	7.3	8.0	6.5	6.2	10.2	7.4
Hyperlipidemia	2.4	3.8	3.8	1.4	1.5	6.5	9.9	3.3
Chronic Hepatitis or Liver Cirrhosis	2.5	1.5	3.1	5.2	1.1	3.6	5.5	3.1
Peptic Ulcer	13.7	14.6	16.1	16.0	7.2	16.8	18.8	14.1
Biliary Stone	2.7	2.8	2.2	3.3	2.8	2.5	4.3	2.9
Cancer	1.5	0.9	1.3	2.8	1.2	0.8	1.3	1.5
Stomach	0.3	0.5	0.4	0.8	0.1	0.2	0.5	0.4
Lung	0.1	0.1	0.0	0.1	0.1	0.1	0.0	0.1
Colon and Rectum	0.3	0.2	0.2	0.3	0.2	0.2	0.3	0.3
Liver	0.3	0.1	0.1	0.9	0.4	0.1	0.0	0.3
Breast	0.0	0.0	0.0	0.0	0.0	0.0	0.0	0.0
Uterus	-	-	-	-	-	-	-	-
Other	0.5	0.0	0.4	0.6	0.5	0.3	0.4	0.5

**Table 2b.** Prevalence of medical history of chronic disease in cohort II females (%).

	Kasama	Kashiwa zaki	Tosa yamada	Arikawa	Miyako	Suita 1	Suita 2	Total
N	9,559	1,645	3,996	6,216	5,269	3,582	2,389	32,656
Stroke	0.7	0.4	0.4	0.4	0.3	0.1	0.9	0.5
Myocardial Infarction	0.8	0.5	0.7	0.9	0.5	0.3	0.7	0.7
Hypertension	18.6	21.5	15.8	21.1	16.6	7.1	17.7	17.2
Diabetes Mellitus	3.9	2.7	3.6	3.7	3.1	1.5	4.2	3.4
Hyperlipidemia	1.9	2.7	3.5	1.1	0.8	3.1	9.0	2.5
Chronic Hepatitis or Liver Cirrhosis	1.3	0.3	1.3	1.8	0.5	0.9	2.2	1.3
Peptic Ulcer	6.1	6.1	7.6	6.4	3.4	6.5	8.7	6.1
Biliary Stone	2.9	2.9	2.4	4.2	3.8	2.6	4.1	3.3
Cancer	2.8	1.5	3.0	3.0	2.5	1.9	3.1	2.7
Stomach	0.2	0.1	0.2	0.3	0.1	0.0	0.1	0.2
Lung	0.1	0.0	0.1	0.1	0.2	0.0	0.0	0.1
Colon and Rectum	0.1	0.1	0.1	0.2	0.1	0.1	0.1	0.1
Liver	0.1	0.1	0.1	0.4	0.1	0.0	0.0	0.1
Breast	0.8	0.5	0.8	0.5	0.2	0.7	1.1	0.6
Uterus	0.7	0.4	1.6	1.2	0.9	0.8	1.4	1.0
Other	0.8	0.4	0.3	0.4	0.9	0.4	0.4	0.6

#### 2) Family History

Regarding the family history of parents in cohort I (Table 3a,b), hypertension was most prevalent in both males and females in every district. A family history of cancer and stroke followed hypertension. Myocardial infarction and angina pectoris were rare. The prevalence of stroke was high in Ninohe, Yokote, and Saku, and low in Ishikawa in both males and females. The prevalence of cancer was high in Katsushika and Saku, and low in Ishikawa and Ninohe in both sexes. The prevalence of stomach cancer was high in Saku and Katsushika and low in Ishikawa. The prevalence of lung cancer was high in Katsushika and low in Ishikawa. A history of myocardial infarction and angina pectoris was more common in Katsushika and Saku and less common in Ishikawa.

**Table 3a.** Prevalence of family history of chronic disease among the parents in cohort I males (%).

	Ninohe	Yokote	Saku	Ishikawa	Katsushika	Total
N	4,228	5,471	5,410	5,156	2,764	23,029
Father						
Stroke	14.8	14.5	11.2	2.3	9.6	10.5
Ischemic Heart Disease	4.0	4.6	5.0	2.4	6.6	4.3
Hypertension	11.1	14.6	14.8	8.1	13.0	12.4
Diabetes Mellitus	5.3	3.7	3.0	1.9	6.7	3.8
Chronic Hepatitis or Liver Cirrhosis	1.2	1.0	1.3	0.7	2.0	1.2
Peptic Ulcer	4.8	4.3	6.0	3.0	5.6	4.6
Cancer	6.1	12.2	15.2	5.3	15.5	10.6
Stomach	1.8	5.6	7.1	1.1	6.3	4.3
Lung	0.9	1.6	1.4	1.2	2.0	1.4
Colon and Rectum	0.5	0.3	0.6	0.2	1.7	0.6
Liver	0.4	0.7	0.8	0.3	0.9	0.6
Mother						
Stroke	8.0	7.3	7.8	2.0	4.5	6.0
Ischemic Heart Disease	2.1	3.2	4.3	1.7	4.2	3.0
Hypertension	15.8	16.7	19.9	11.7	14.5	15.9
Diabetes Mellitus	5.0	4.8	4.7	2.8	6.1	4.6
Chronic Hepatitis or Liver Cirrhosis	0.5	0.6	0.9	0.4	1.2	0.7
Peptic Ulcer	2.2	2.0	2.9	1.4	2.1	2.1
Cancer	4.4	7.9	9.5	5.2	11.2	7.4
Stomach	0.9	2.9	3.0	0.5	3.3	2.1
Lung	0.4	0.5	0.6	0.4	0.6	0.5
Colon and Rectum	0.3	0.5	0.5	0.3	1.2	0.5
Liver	0.3	0.3	0.4	0.2	0.5	0.4
Brest	0.5	0.5	0.8	0.2	1.2	0.6

**Table 3b.** Prevalence of family history of chronic disease among the parents in cohort I females (%).

	Ninohe	Yokote	Saku	Ishikawa	Katsushika	Total
N	4,873	6,283	5,477	5,297	3,984	25,914
Father						
Stroke	13.9	14.4	12.0	1.9	8.1	10.3
Ischemic Heart Disease	3.0	4.2	5.3	1.9	6.4	4.1
Hypertension	9.2	11.3	12.9	7.7	15.0	11.1
Diabetes Mellitus	4.2	2.8	3.2	1.9	6.8	3.6
Chronic Hepatitis or Liver Cirrhosis	0.6	0.8	1.2	0.5	1.9	0.9
Peptic Ulcer	3.4	3.8	5.2	3.0	5.4	4.1
Cancer	6.2	11.0	15.6	5.0	17.0	10.8
Stomach	1.9	5.4	7.0	0.8	6.9	4.4
Lung	1.0	1.1	1.6	1.0	2.6	1.4
Colon and Rectum	0.5	0.2	0.4	0.3	1.4	0.5
Liver	0.6	0.8	1.2	0.4	1.6	0.9
Mother						
Stroke	8.4	8.3	8.7	1.6	4.0	6.4
Ischemic Heart Disease	2.4	4.0	4.8	1.5	5.1	3.5
Hypertension	13.2	15.1	19.8	13.4	18.5	15.9
Diabetes Mellitus	3.9	4.5	4.5	3.0	7.0	4.5
Chronic Hepatitis or Liver Cirrhosis	0.5	0.5	0.8	0.3	1.1	0.6
Peptic Ulcer	2.2	2.1	2.4	1.6	3.0	2.2
Cancer	4.8	7.3	11.3	4.1	11.3	7.6
Stomach	1.2	2.7	3.7	0.4	3.2	2.2
Lung	0.3	0.6	0.8	0.4	1.1	0.6
Colon and Rectum	0.3	0.3	0.5	0.1	1.2	0.4
Liver	0.3	0.5	0.8	0.2	0.7	0.5
Brest	0.4	0.4	0.7	0.3	1.0	0.5

In cohort II (Table 4a,b), the prevalence of family history of both stroke and cancer was high in Kasama and Kashiwazaki in males and females; Suita subcohorts 1 and 2 only showed a higher prevalence of cancer. A history of stomach cancer was most common, followed by lung, colon, and liver cancer. In Miyako, the prevalence of stroke, cancer, and heart disease was the lowest among all districts.

**Table 4a.** Prevalence of family history of chronic disease among the parents in cohort II males (%).

	Kasama	Kashiwa zaki	Tosa yamada	Arikawa	Miyako	Suita 1	Suita 2	Total
N	9,453	1,520	3,561	4,983	4,779	2,907	2,094	29,297
Stroke	21.1	25.4	18.2	13.8	9.6	12.1	18.7	16.8
Diabetes	8.4	5.7	7.9	6.0	4.7	11.7	9.5	7.6
Heart Disease	10.2	9.5	10.2	9.6	3.2	11.0	12.4	9.2
Cancer	20.8	21.3	21.2	16.4	9.1	25.9	26.0	19.1
Stomach	5.4	4.8	6.1	3.4	1.9	7.3	8.9	5.0
Lung	1.4	1.0	1.7	1.0	1.0	3.0	2.2	1.5
Colon and Rectum	1.4	1.4	1.8	0.5	0.3	2.3	2.2	1.3
Liver	0.9	0.5	0.8	1.3	0.5	2.1	1.6	1.0
Breast	0.3	0.3	0.5	0.4	0.2	0.7	0.8	0.4
Uterus	0.7	0.5	1.2	0.8	0.3	1.2	1.2	0.8
Pancreas	0.6	0.7	0.7	0.4	0.1	1.1	0.9	0.6
Others and Multiple	2.8	3.0	2.7	2.0	1.3	5.1	6.3	2.9
Unidentified	7.1	9.1	5.6	6.6	3.5	3.2	1.9	5.6

**Table 4b.** Prevalence of family history of chronic disease among the parents in cohort II females (%).

	Kasama	Kashiwa zaki	Tosa yamada	Arikawa	Miyako	Suita 1	Suita 2	Total
N	9,559	1,645	3,996	6,216	5,239	3,582	2,389	32,626
Stroke	21.1	24.9	17.6	12.7	8.2	10.8	17.0	15.7
Diabetes	7.4	4.0	8.5	4.7	4.4	12.4	10.9	7.2
Heart Disease	12.1	10.9	11.6	9.4	3.6	12.3	13.7	10.3
Cancer	19.4	22.5	19.5	15.6	8.4	26.1	30.6	18.6
Stomach	5.8	6.0	5.9	3.1	1.6	7.2	9.9	5.1
Lung	1.4	0.8	1.5	1.1	0.9	2.3	3.2	1.5
Colon and Rectum	1.2	1.1	1.1	0.9	0.4	2.2	2.1	1.2
Liver	1.0	0.8	1.0	1.1	0.4	1.9	2.2	1.1
Breast	0.4	0.5	0.9	0.2	0.2	0.9	0.6	0.5
Uterus	0.9	0.7	1.4	0.6	0.5	1.6	2.0	1.0
Pancreas	0.4	0.4	0.8	0.3	0.2	1.0	1.0	0.5
Others and Multiple	2.7	3.0	3.1	2.2	1.6	6.6	7.4	3.3
Unidentified	5.6	9.2	4.0	6.2	2.6	2.4	2.2	4.6

#### 3) Past History of Blood Transfusion

Past history of blood transfusion was about 10% in every district. It was low in Katsusika (6.8%) and Suita subcohort 1 (6.2%) in males, and high in Saku females (17.6%).

#### 4) Medical Treatment

The frequency of visiting physicians to receive any medication was 17.6% to 27.9% in males and 18.9% to 30.4% in females in cohort I (Table 5a). Yokote showed a high frequency in males and females, whereas Ishikawa and Saku showed a low frequency. The most common medication was for hypertension, 39.8% to 52.6% in males and 45.4% to 48.7% in females. The frequency of medication for hypercholesterolemia was 4.3% to 9.9% in males and 3.8% to 13.9% in females; low frequencies were seen in Saku and Ishikawa and high frequencies in Ninohe and Yokote. Medication for diabetes mellitus was reported for 6.6% to 10.7% of the males and 4.0% to 7.0% of the females; the rates were highest in Ishikawa.

**Table 5a.** Proportion of medicated participants and proportion of medicated diseases in cohort I .

	Ninohe	Yokote	Saku	Ishikawa	Katsushika	Total
Male						
Proportion of medicated participants						
	25.1%	27.9%	21.4%	17.6%	16.1%	22.1%
	(1,049)	(1,524)	(1,129)	(906)	(441)	(5,049)
Proportion of medicated diseases among medicated participants						
Hypertension	49.7%	52.6%	46.7%	39.8%	30.8%	46.5%
Hypercholesterolemia	9.9%	8.2%	4.3%	6.1%	9.3%	7.4%
Diabetes	10.7%	6.6%	6.7%	10.5%	7.7%	8.3%
Gout	3.8%	2.8%	5.4%	6.2%	9.3%	4.8%
Others	33.4%	33.9%	40.7%	39.6%	54.9%	38.1%
Unknown	6.1%	6.8%	7.1%	7.1%	2.5%	6.4%

Female						
Proportion of medicated participants						
	28.8%	30.4%	22.2%	18.9%	15.3%	23.7%
	(1,389)	(1,907)	(1,181)	(998)	(606)	(6,081)
Proportion of medicated diseases among medicated participants						
Hypertension	45.9%	45.4%	48.7%	45.7%	32.4%	44.9%
Hypercholesterolemia	10.1%	13.9%	3.8%	4.7%	6.3%	8.8%
Diabetes	4.0%	4.2%	4.2%	7.0%	2.0%	4.4%
Gout	2.2%	1.4%	1.0%	1.7%	0.2%	1.4%
Others	40.2%	36.8%	42.7%	43.9%	63.0%	42.5%
Unknown	7.1%	8.6%	7.3%	5.1%	1.8%	6.8%

In cohort II, the frequency of visiting physicians and taking medication increased to 29.0% to 38.9% for males and 29.3% to 34.9% for females (Table 5b). The Suita subcohort 2 showed a high frequency in both males and females, whereas the rate in Suita subcohort 1 was less than 20%.

**Table 5b(old 6b8ab).** Proportion of medicated participants and proportion of medicated diseases in cohort II .

	Kasama	Kashiwa zaki	Tosa yamada	Arikawa	Miyako	Suita 1	Suita 2	Total
Male								
Proportion of medicated participants								
	31.8%	36.4%	30.3%	34.0%	29.0%	19.4%	38.9%	31.0%
	(2,997)	(550)	(1,077)	(1,691)	(1,375)	(560)	(815)	(9,065)
Proportion of medicated diseases among medicated participants								
Hypertension	50.1%	57.3%	43.9%	43.8%	54.5%	27.9%	38.8%	46.9%
Hyperlipidemia	5.6%	4.0%	6.5%	3.9%	1.5%	9.3%	13.1%	5.6%
Diabetes	9.2%	3.5%	8.3%	9.6%	9.6%	7.5%	10.8%	8.9%
Gout	5.2%	2.2%	10.0%	5.0%	8.7%	10.7%	7.6%	6.6%
Angina	5.3%	3.3%	7.1%	6.6%	3.6%	2.0%	7.0%	5.3%
Unidentified	3.6%	4.7%	7.2%	7.4%	2.8%	3.8%	6.4%	4.9%
Others	29.8%	27.6%	35.7%	33.5%	26.1%	47.0%	44.9%	32.9%

Female								
Proportion of medicated participants								
	32.6%	34.7%	29.8%	34.8%	29.3%	15.5%	34.9%	30.6%
	(3,110)	(566)	(1,188)	(2,158)	(1,534)	(554)	(833)	(9,943)
Proportion of medicated diseases among medicated participants								
Hypertension	54.2%	59.7%	43.6%	53.2%	52.3%	23.8%	38.7%	49.8%
Hyperlipidemia	8.3%	5.5%	9.3%	4.9%	2.5%	6.0%	19.0%	7.4%
Diabetes	5.9%	3.4%	5.6%	5.1%	7.2%	2.7%	4.7%	5.4%
Gout	0.9%	0.7%	0.8%	2.7%	1.4%	1.1%	0.6%	1.3%
Angina	4.1%	3.7%	4.5%	5.5%	3.4%	2.9%	4.7%	4.3%
Unidentified	3.2%	3.9%	5.7%	6.3%	1.8%	3.2%	4.9%	4.1%
Others	29.9%	27.6%	42.0%	28.3%	32.7%	64.6%	50.4%	34.9%

The frequency of medication for hypertension was also common: 38.8% to 57.3% in males and 38.7% to 59.7% in females. This trend was the same in cohort I, except for low treatment rate for hypertension in Suita and Tosayamada.

The rates of medication for hyperlipidemia were 1.5% to 13.1% in males and 2.5% to 19.0% in females, with lower rates in Arikawa and Miyako and higher rates in Suita subcohort 2. Medication for diabetes mellitus was reported by 3.5% to 10.8% of the males and 3.4% to 7.2% of the females. No significant differences among districts were noted except for Kashiwazaki, which showed a lower frequency.

#### 5) Intake of Vitamin Supplements

In cohort I, about 15% of both male and female participants took vitamin compound tablets at least once per week in every district. Use of multivitamin compounds was more common among males, whereas vitamin C and vitamin E tablets were popular among females.

In cohort II, 15% of the male and 20% of the female took vitamin compound tablets at least once per week. A high prevalence of vitamin use was found in Suita subcohort 2. Multivitamin compounds were more commonly taken by males, whereas vitamin C and vitamin E were more commonly taken by females, as in cohort I.

#### 6) Basic Health Checkup by Mass Screening Program

In cohort I (Table 6a,b), the participation rate for examination of blood pressure, blood chemistry, and chest x-rays by the local government was greater than 70% in all districts. Katsushika was low because of different selection method of participants. The participation rate for electrocardiographic and funduscopic examinations was 50% to 60% in Ninohe, Yokote, and Saku where the prevalence of hypertension has been higher than other districts. Participation rate for x-ray examination of the stomach was high in Yokote and Saku (about 40% in males and females). For gastrointestinal endoscopy, the participation rate was the highest in Saku (36% for males and 30% for females). Occult blood stool examination was also frequently conducted in Saku (33% for males and 39% for females). Moreover, Saku showed a higher participation rate for colon endoscopy than the other districts.

**Table 6a.** Participation rate of various health examination in cohort I males (%).

	Ninohe	Yokote	Saku	Ishikawa	Katsushika	Total
N	4,228	5,471	5,410	5,156	2,764	23,029
Blood Pressure	78.2	81.7	71.8	76.0	39.2	72.4
Blood Test	59.0	69.3	62.7	69.6	33.8	61.7
ECG	56.3	63.6	57.1	41.2	27.2	51.3
Funduscopy	45.7	39.4	45.1	29.5	10.2	36.2
Chest X-Ray	73.8	75.1	67.0	72.8	35.1	67.6
Cytodiagnostic of Sputum	5.4	5.9	13.0	8.7	1.8	7.6
Barium Examination of Stomach	29.9	47.2	40.0	31.2	14.3	34.8
Gastrointestinal Endoscopy	10.1	16.4	36.4	8.3	3.2	16.6
Occult Blood in Stool	9.5	14.1	33.5	11.1	3.6	15.9
Barium Examination of Colon	3.1	3.8	9.4	4.2	1.1	4.8
Colon Endoscopy	2.5	5.0	14.7	2.9	0.7	5.8
Unknown	0.3	0.0	0.0	0.1	0.0	0.1

**Table 6b.** Participation rate of various health examination in cohort I females (%).

	Ninohe	Yokote	Saku	Ishikawa	Katsushika	Total
N	4,873	6,283	5,477	5,297	3,984	25,914
Blood Pressure	81.5	80.8	76.7	74.1	33.3	71.4
Blood Test	65.3	70.3	69.4	69.2	30.3	62.8
ECG	64.8	60.7	65.1	34.8	17.7	50.5
Funduscopy	55.4	44.8	51.0	23.9	7.9	38.1
Chest X-Ray	79.1	73.9	72.6	69.0	28.0	66.5
Cytodiagnostic of Sputum	3.0	1.7	6.6	3.6	0.9	3.2
Barium Examination of Stomach	28.5	39.9	44.2	26.0	12.5	31.6
Gastrointestinal Endoscopy	8.2	12.2	30.3	6.1	1.9	12.4
Occult Blood in Stool	8.4	11.7	39.0	8.6	2.5	14.8
Barium Examination of Colon	3.4	3.7	9.3	3.3	0.7	4.3
Colon Endoscopy	2.3	3.4	13.8	1.9	0.3	4.6
Unknown	0.1	0.0	0.0	0.0	0.1	0.1

In cohort II (Table 7a,b), the participation rate for basic health examination within the latest year was more than 70% in every district, except Suita.

**Table 7a.** Participation rate of various health examination within 1 year in cohort II males (%).

	Kasama	Kashiwa zaki	Tosa yamada	Arikawa	Miyako	Suita 1	Suita 2	Total
Health Examination	80.7	92.3	78.5	73.0	77.6	79.5	78.0	78.9
Measurement of Blood Pressure	87.6	96.3	87.1	84.3	86.0	87.6	89.8	87.3
Measurement of Serum Cholesterol	54.1	52.6	54.1	34.8	46.4	63.0	70.9	52.0

**Table 7b.** Participation rate of various health examination within 1 year in cohort II females (%).

	Kasama	Kashiwa zaki	Tosa yamada	Arikawa	Miyako	Suita 1	Suita 2	Total
Health Examination	81.4	91.3	80.4	72.9	80.0	57.5	68.5	76.3
Measurement of Blood Pressure	89.6	95.4	90.3	84.3	89.1	72.7	85.0	86.6
Measurement of Serum Cholesterol	58.6	58.9	61.3	38.7	51.5	47.7	63.5	53.4

More than 85% of participants had blood pressure measurements in every district. The frequency of participants in serum cholesterol measurement was high in Suita subcohort 2 (70.9% in males and 63.5% in females) and low in Arikawa (34.8% in males and 38.7% in females). The other districts showed almost the same frequency, 50% in both males and females.

For participants who received the basic health examination in cohort II, we asked how much they recognized their results of the health examination (Table 8a,b). The proportion of participants who recognized their own value of systolic blood pressure among those who measured blood pressure was 64.6% to 83.6% of the males and 72.4% to 82.5% of the females; the highest proportion was in Suita subcohort 2 in both males and females and the lowest in Arikawa participants. A similar result was found for diastolic blood pressure.

**Table 8a.** Recognition rate* of blood pressure and serum cholesterol Value in cohort II males (%).

	Kasama	Kashiwa zaki	Tosa yamada	Arikawa	Miyako	Suita 1	Suita 2	Total
Systolic Blood Pressure	76.9	76.9	69.8	64.6	73.4	69.4	83.6	73.3
Diastolic Blood Pressure	76.5	76.4	69.2	63.8	73.0	68.4	83.1	72.7
Seram Cholesterol	33.4	28.4	37.0	31.3	25.6	34.7	56.8	35.0

* proportion of participants who recognized their own value among those who measured blood pressure and serum cholesterol.

**Table 8b.** Recognition rate* of blood pressure and serum cholesterol Value in cohort II females (%).

	Kasama	Kashiwa zaki	Tosa yamada	Arikawa	Miyako	Suita 1	Suita 2	Total
Systolic Blood Pressure	79.5	78.5	76.4	72.4	79.6	68.8	82.5	76.9
Diastolic Blood Pressure	78.9	77.8	75.4	71.7	79.0	67.1	80.9	76.1
Seram Cholesterol	46.3	36.9	51.2	45.8	34.6	56.5	69.6	48.1

* proportion of participants who recognized their own value among those who measured blood pressure and serum cholesterol.

The proportion of participants who recognized their own serum cholesterol values among males was the lowest in Miyako (25.6%) and the highest in Suita subcohort 2 (56.8%). In females, the lowest proportion was in Miyako (34.6%) and the highest was in the Suita subcohort 2 (69.6%). In other districts, the proportion of participants who recognized their own serum cholesterol values was under 50%.

#### 7) Change of Body Weight from Age 20

In cohort I, the proportion of participants who gained more than 5 kg was compared with the body weight at the age of 20. Ishikawa participants showed the highest proportion in both males (71.6%) and females (74.4%). It was 46% to 49% in males and 54% to 56% in females in other districts of cohort I (Table 9a,b). Mean body weight compared with body weight at 20 years old increased in every district in cohort II, in both males and females; the increase was larger in men in Suita subcohorts 1 and 2 and in both males and females in Miyako (Table 10a,b).

**Table 9a.** Proportion of participants who changed over 5kg in body weight compared with one-self at 20years old in cohort I males (%).

	Ninohe	Yokote	Saku	Ishikawa	Katsushika	Total
N	4,171	5,381	5,315	5,129	2,757	22,753
Decrease	16.0	15.3	12.4	7.3	6.0	11.8
No Change	37.6	37.9	38.5	21.1	28.5	33.1
Increase	46.4	46.9	49.0	71.6	65.5	55.1

**Table 9b.** Proportion of participants who changed over 5kg in body weight compared with one-self at 20years old in cohort II females (%).

	Ninohe	Yokote	Saku	Ishikawa	Katsushika	Total
N	4,825	6,162	5,403	5,287	3,973	25,650
Decrease	17.5	16.3	16.6	9.2	9.8	14.1
No Change	28.9	28.6	27.7	16.5	31.8	26.5
Increase	53.6	55.1	55.8	74.4	58.4	59.4

**Table 10a.** Mean body weight at present and aged 20 years old in cohort II males (kg).

	Kasama	Kashiwa zaki	Tosa yamada	Arikawa	Miyako	Suita 1	Suita 2	Total
Current	63.2 ± 9.0	59.7 ± 8.3	62.7 ± 9.3	63.1 ± 9.0	64.4 ± 9.6	65.5 ± 8.9	63.3 ± 8.9	63.3 ± 9.2
20 Years old	58.7 ± 7.0	59.5 ± 6.9	59.5 ± 8.8	60.9 ± 8.2	58.9 ± 7.0	58.8 ± 7.2	58.2 ± 7.1	59.2 ± 8.0

**Table 10b.** Mean body weight at present and aged 20 years old in cohort II females (kg).

	Kasama	Kashiwa zaki	Tosa yamada	Arikawa	Miyako	Suita 1	Suita 2	Total
Current	54.0 ± 7.6	52.1 ± 7.2	53.3 ± 7.7	54.8 ± 8.6	54.8 ± 8.4	53.1 ± 7.1	52.9 ± 7.8	53.9 ± 7.9
20 Years old	49.5 ± 6.0	49.9 ± 6.0	49.0 ± 5.9	51.1 ± 8.9	48.7 ± 6.6	48.7 ± 5.5	48.6 ± 6.1	49.4 ± 6.6

#### 8) Body Mass Index (BMI)

The prevalence of BMI in both sexes is shown in Tables 11a,b and 12a,b. BMI was over 26.0 in almost one third of the participants in Ishikawa and Miyako.

**Table 11a.** Body Mass Index in cohort I males (%).

		Ninohe	Yokote	Saku	Ishikawa	Katsushika
	N	4,170	5,469	5,406	5,119	2,741
BMI						
	-17	1.0	1.3	1.6	1.0	1.4
	18-19	7.1	8.2	9.6	5.3	8.8
	20-21	20.9	23.2	23.9	14.3	20.5
	22-23	29.6	31.4	28.7	25.0	27.6
	24-25	24.7	22.9	22.8	26.6	23.4
	26-	16.9	13.0	13.5	27.7	18.3

	mean	23.1	22.7	22.7	24.0	23.0
	S.D.	2.8	2.6	2.7	3.3	2.9

**Table 11b.** Body Mass Index in cohort I Females (%).

		Ninohe	Yokote	Saku	Ishikawa	Katsushika
	N	4,812	6,278	5,474	5,279	3,928
	-17	1.7	2.1	1.9	1.4	3.0
BMI						
	18-19	7.9	10.3	10.5	6.3	13.7
	20-21	20.7	23.3	23.3	17.1	26.8
	22-23	26.9	27.8	27.1	25.6	25.4
	24-25	22.0	19.4	21.2	21.8	16.7
	26-	20.8	17.1	16.1	27.7	14.4

	mean	23.2	22.8	22.7	23.9	22.3
	S.D.	3.2	3.1	2.9	3.7	3.1

**Table 12a.** Body Mass Index in cohort II males (%).

		Kasama	Kashiwa zaki	Tosa yamada	Arikawa	Miyako	Suita 1	Suita 2
	N	9,511	1,504	3,521	5,000	4,893	2,908	2,090
BMI								
	-17	2.0	1.9	2.8	1.9	0.8	2.0	3.3
	18-19	8.7	12.6	10.5	8.8	3.8	9.6	11.0
	20-21	21.7	29.1	23.7	21.8	12.3	22.7	22.0
	22-23	30.2	28.9	27.2	29.1	25.1	29.2	29.7
	24-25	21.9	17.8	20.5	21.5	26.9	22.4	21.0
	26-	15.5	9.8	15.2	16.8	31.2	14.1	13.1

	mean	23.3	22.6	23.1	23.4	24.8	23.2	23.0
	S.D.	2.8	2.6	2.9	2.9	3.1	2.7	2.8

**Table 12b.** Body Mass Index in cohort II Females (%).

		Kasama	Kashiwa zaki	Tosa yamada	Arikawa	Miyako	Suita 1	Suita 2
	N	9,727	1,619	4,034	6,237	5,415	3,568	2,389
BMI								
	-17	2.4	2.6	3.4	2.5	1.4	3.9	4.1
	18-19	9.5	10.8	12.1	9.7	6.1	20.2	15.4
	20-21	23.2	23.2	24.2	20.5	16.9	32.3	28.1
	22-23	26.9	30.1	25.1	24.9	26.2	23.0	26.7
	24-25	20.0	18.8	18.7	20.4	22.4	12.3	14.4
	26-	18.1	14.5	16.5	22.0	27.0	8.4	11.3

	mean	23.4	23.0	23.1	23.7	24.3	22.0	22.5
	S.D.	3.1	2.9	3.2	3.5	3.5	2.8	3.1

### Life Habits

#### 1) Opportunities to Participate in Sports and Physical Exercise

In every district of cohorts I and II, the proportion of participants who responded “almost never” to participation in sports and physical exercise was high in both sexes. Generally, it was higher in females (around 75%) than in males (65%). In Saku, Katsushika, Ishikawa, Miyako, and Suita subcohort 2, the proportion of participants who did sports and physical exercise once or more per week was higher in both sexes than in other districts (Tables 13a,b and 14a,b). In cohort II, the proportion of participants who engaged in physical labor or intensive sports for 1 hour or more per day was 40% to 50% in all districts except Suita subcohort 2 (around 20%) (Table 15a,b).

**Table 13a.** Proportion of participants who did sports and physical exercise in cohort I males (%).

	Ninohe	Yokote	Saku	Ishikawa	Katsushika	Total
N	4,199	5,416	5,326	5,150	2,762	22,853
Almost never	75.8	68.3	56.0	65.5	62.8	65.5
1-3 days a month	12.0	16.3	25.1	11.7	17.9	16.7
1-2 days a week	5.8	8.4	12.1	10.8	12.5	9.8
3-4 days a week	2.5	3.2	3.6	6.4	4.2	4.0
Almost everyday	3.9	3.8	3.3	5.6	2.6	4.0

**Table 13b.** Proportion of participants who did sports and physical exercise in cohort I males (%).

	Ninohe	Yokote	Saku	Ishikawa	Katsushika	Total
N	4,815	6,150	5,380	5,291	3,973	25,609
Almost never	86.1	81.7	68.1	78.9	66.4	76.7
1-3 days a month	4.9	6.2	11.7	5.0	7.1	7.0
1-2 days a week	3.9	6.4	10.9	8.5	18.0	9.1
3-4 days a week	2.0	2.5	4.7	3.5	5.0	3.5
Almost everyday	3.2	3.3	4.6	4.1	3.4	3.7

**Table 14a.** Proportion of participants who did sports and physical exercise in cohort II males (%).

	Kasama	Kashiwa zaki	Tosa yamada	Arikawa	Miyako	Suita 1	Suita 2	Total
N	9,386	1,498	3,538	4,958	4,724	2,898	2,090	29,092
Almost never	6.4	77.7	72.2	73.2	59.2	53.3	52.9	64.7
1-3 days a month	16.7	10.5	11.5	9.2	14.5	26.3	17.1	15.1
1-2 days a week	9.4	6.3	8.4	6.3	10.1	14.9	16.1	9.7
3-4 days a week	4.2	2.1	3.4	4.4	7.8	3.4	6.3	4.7
Almost everyday	5.6	3.4	4.5	6.9	8.4	2.1	7.6	5.8

**Table 14b.** Proportion of participants who did sports and physical exercise in cohort II females (%).

	Kasama	Kashiwa zaki	Tosa yamada	Arikawa	Miyako	Suita 1	Suita 2	Total
N	9,464	1,631	3,962	6,172	5,225	3,560	2,386	32,400
Almost never	74.6	79.2	81.2	75.4	64.6	64.9	61.7	72.2
1-3 days a month	7.3	6.9	4.6	5.3	9.6	10.9	7.7	7.4
1-2 days a week	8.8	6.4	6.7	9.2	10.3	17.4	16.5	10.3
3-4 days a week	4.1	3.4	3.1	4.2	7.6	4.3	7.6	4.8
Almost everyday	5.3	4.1	4.3	5.8	7.9	2.6	6.5	5.4

**Table 15a.** Proportion of participants who was engaged in physical labor or intensive sports in cohort II males (%).

	Kasama	Kashiwa zaki	Tosa yamada	Arikawa	Miyako	Suita 1	Suita 2	Total
N	9,326	1,481	3,503	4,946	4,724	2,849	2,094	28,923
none	41.0	27.6	42.0	34.4	33.9	57.7	66.7	41.7
less than 1 hour	17.5	11.6	14.4	12.6	14.9	18.8	12.8	15.4
more then 1 hour	41.5	60.8	43.5	53.0	51.1	23.6	20.5	43.0

**Table 15b.** Proportion of participants who was engaged in physical labor or intensive sports in cohort II females (%).

	Kasama	Kashiwa zaki	Tosa yamada	Arikawa	Miyako	Suita 1	Suita 2	Total
N	9,338	1,602	3,910	6,144	5,180	3,496	2,385	32,055
none	54.1	48.1	55.0	48.1	44.7	67.0	77.8	54.4
less than 1 hour	14.1	12.3	11.4	9.9	12.8	13.0	6.4	11.9
more then 1 hour	31.9	39.6	33.6	42.0	42.5	20.0	15.8	33.6

#### 2) Hours of Sleep and Work

The mean number of hours of sleep was short in both sexes in urban districts such as Katsushika (7.1 hours for males and 6.8 hours for females), Suita subcohort 1 (6.9 hours for males and 6.7 hours for females), and subcohort 2 (7.1 hours for males and 6.8 hours for females) in comparison with other districts (about 7.5 hours for males and 7.1 hours for females). In Ishikawa, the mean was low as well as urban districts. In Katsushika, Suita subcohorts 1 and 2, and Ishikawa, the proportion who averaged 6 to 7 hours of sleep was high. When males and females were compared, mean number of sleeping hours in females was less than that for males in all districts. Among males, the proportion who had 9 hours or more sleeping was high in all rural districts (10% or more) except for Katsushika, Suita, and Ishikawa (Tables 16a,b and 17a,b).

**Table 16a.** Distribution of sleeping hour in cohort I males (%).

	Ninohe	Yokote	Saku	Ishikawa	Katsushika	Total
N	4,169	5,338	5,306	5,155	2,762	22,730
less than 6 hours	1.8	2.2	2.2	6.4	3.6	3.3
6-7 hours	8.6	12.1	10.7	22.4	22.4	14.7
7-8 hours	28.2	33.5	33.6	34.2	40.2	33.5
8-9 hours	47.3	42.9	43.8	31.9	30.6	39.9
more than 9 hours	14.1	9.2	9.6	5.2	3.2	8.6

mean sleeping hour	7.7	7.5	7.5	7.1	7.1	7.4
SD	1.0	1.0	1.0	1.1	0.9	1.0

**Table 16b.** Distribution of sleeping hour in cohort I females (%).

	Ninohe	Yokote	Saku	Ishikawa	Katsushika	Total
N	4,805	6,094	5,359	5,291	3,979	25,528
less than 6 hours	3.2	3.2	3.6	11.7	5.1	5.3
6-7 hours	13.7	18.3	18.9	28.0	28.8	21.2
7-8 hours	36.5	40.0	41.4	33.5	46.0	39.2
8-9 hours	39.0	33.4	31.7	24.1	18.7	29.9
more than 9 hours	7.6	5.0	4.2	2.6	1.3	4.4

mean sleeping hour	7.4	7.2	7.1	6.8	6.8	7.1
SD	1.0	1.0	0.9	1.1	0.9	1.0

**Table 17a(old 20 a).** Distribution of sleeping hour in cohort II males (%).

	Kasama	Kashiwa zaki	Tosa yamada	Arikawa	Miyako	Suita 1	Suita 2	Total
N	9,292	1,482	3,532	4,802	4,280	2,896	2,068	28,352
less than 6 hours	3.1	2.1	3.7	7.1	4.1	4.1	4.8	4.2
6-7 hours	14.4	13.2	13.9	16.8	14.6	26.9	22.4	16.6
7-8 hours	31.1	35.2	28.6	26.1	29.7	43.3	38.9	31.8
8-9 hours	38.5	38.8	39.2	36.4	38.5	22.6	28.4	35.9
more than 9 hours	12.9	10.6	14.7	13.8	13.0	3.0	5.4	11.7

mean sleeping hour	7.5	7.4	7.5	7.4	7.5	6.9	7.1	7.4
SD	1.2	1.0	1.2	1.3	1.3	0.9	1.1	1.2

**Table 17b(old 20 b).** Distribution of sleeping hour in cohort II females (%).

	Kasama	Kashiwa zaki	Tosa yamada	Arikawa	Miyako	Suita 1	Suita 2	Total
N	9,399	1,596	3,973	5,966	4,648	3,572	2,359	31,513
less than 6 hours	4.5	2.6	4.5	5.2	5.9	6.8	7.8	5.2
6-7 hours	20.1	17.9	19.8	17.2	18.3	31.6	31.2	21.3
7-8 hours	36.6	45.8	38.5	32.1	32.8	43.9	39.1	36.9
8-9 hours	31.0	28.8	30.9	36.4	34.6	16.0	19.0	29.8
more than 9 hours	7.8	5.1	6.4	9.2	8.6	1.6	2.7	6.8

mean sleeping hour	7.2	7.2	7.2	7.3	7.2	6.7	6.8	7.1
SD	1.1	0.9	1.0	1.2	1.2	0.9	1.0	1.1

For working hours, in cohort II Kashiwazaki and Suita subcohort 1 showed longer working hours in both sexes than other districts. Mean working hours in Kashiwazaki was 9.2 hours for males and 10.0 hours for females. A mode of working hours was 10 to 11 hours in both males and females. Mean working hours in Suita subcohort 1 was 9.5 hours for males and 9.0 hours for females with a mode of 10 to 11 hours in both sexes. In Suita subcohort 1, working hours were long and sleeping hours were short in comparison with other districts.

#### 3) Bowel Movement

In cohorts I and II, females showed a higher prevalence of constipation than males. In cohort I, the proportion of irregular bowel movements was about 30% in females and 10% in males in every district. In cohort II, the proportion of 2 to 3 times per week was about 20% in females and 10% in males. There were no differences among the districts in the frequency of bowel movements.

#### 4) Personality

The prevalence of “easygoing” persons was high in Ishikawa and Miyako (around 18% in both sexes) in comparison with other districts. On the other hand, the prevalence of “impetuous” persons was high in urban districts such as Katsushika (38% in males and 31% in females) and Suita subcohort 1 (41% in males and 31% in females) and subcohort 2 (39% in males and 29% in females).

### Social Background

#### 1) Area of Residence

Approximately 90% of the participants of both sexes in cohort I and more in cohort II reported that they had been living in the same residence as his or her place of birth and residence at age 20. Urban residents in Katsusika and Suita showed shorter period after settlement. Ishikawa was also low because many soldiers were included.

#### 2) Occupation

It was difficult to compare occupations between cohorts I and II because the classification of occupation was different in the questionnaires. The urban areas of Katsushika and Suita, however, showed very low rates of agriculture, forestry, and fishery. Instead, occupations such as office work, selling, service, and physical labor were dominant. Ishikawa also showed a low rate of agriculture, forestry, and fishery, and there were many transportation and communication workers. Nearly 25% participants in Ninohe, Yokote, Saku, Kashiwazaki, Tosayamada, and Miyako reported agriculture as their occupation. It was characteristic that the fishery business was common for males in Arikawa (Tables 18a,b and 19a,b).

**Table 18a.** Occupation of participants in cohort I males (%).

	Ninohe	Yokote	Saku	Ishikawa	Katsushika	Total
N	4,168	5,459	5,370	5,092	2,746	22,835
Expert	4.9	6.3	5.5	7.1	8.6	6.3
Management	1.7	4.7	2.7	3.6	5.0	3.5
Clerical	11.8	11.7	12.5	11.5	12.9	12.0
Sales	8.3	11.2	7.0	6.4	16.6	9.3
Service	3.2	3.5	4.1	4.6	9.0	4.5
Security	0.3	1.0	0.4	4.0	6.7	2.1
Agriculture,Forestry,Fishery	25.6	26.2	23.4	10.3	1.6	19.0
Transport,Communication	8.4	6.8	6.0	12.2	6.1	8.0
Physical labor	32.4	25.3	36.6	34.1	31.6	32.0
Unclassified	0.0	0.0	0.0	0.0	0.4	0.1
Unemployed	3.4	3.3	1.7	6.2	1.4	3.4

**Table 18b.** Occupation of participants in cohort I females (%).

	Ninohe	Yokote	Saku	Ishikawa	Katsushika	Total
N	4,805	6,245	5,425	5,250	3,919	25,644
Expert	3.9	4.7	5.3	6.5	2.5	4.7
Management	0.2	0.3	0.2	0.5	0.4	0.3
Clerical	6.0	7.2	8.6	9.4	20.6	9.8
Sales	7.9	7.5	6.2	8.4	9.6	7.8
Service	6.4	13.6	9.2	17.6	10.3	11.6
Security	0.1	0.0	0.0	0.1	3.0	0.5
Agriculture,Forestry,Fishery	30.0	20.5	28.7	6.8	1.8	18.3
Transport,Communication	0.5	0.5	0.4	0.3	0.3	0.4
Physical labor	19.8	16.7	26.4	7.6	19.2	17.8
Unclassified	0.0	0.0	0.0	0.0	1.3	0.2
Unemployed	25.2	29.1	15.1	42.9	31.1	28.5

**Table 19a.** Occupation of participants in cohort II males (%).

	Kasama	Kashiwa zaki	Tosa yamada	Arikawa	Miyako	Suita 1	Suita 2	Total
N	9,355	1,509	3,530	4,960	4,745	2,894	2,094	29,087
Agriculture	11.5	29.0	26.0	8.5	28.1	0.0	0.2	14.4
Forestry	0.2	0.4	0.4	0.3	0.1	0.0	0.0	0.2
Fishery	0.0	0.0	0.4	28.3	2.8	0.0	0.0	5.3
Employed	50.8	26.3	43.5	25.3	33.5	78.1	60.7	44.9
Business on one’s own	17.0	11.0	12.5	12.9	15.5	15.1	16.7	15.0
Expert	2.7	1.3	2.1	1.4	3.3	3.7	3.3	2.5
Housewife	0.0	0.0	0.0	0.0	0.0	0.0	0.0	0.0
Unemployed	8.0	2.5	9.1	9.0	7.4	1.2	17.0	7.9
Others	0.5	0.1	0.3	0.9	0.5	0.7	0.5	0.5
Agriculture+Employed	5.8	23.3	3.1	2.5	2.9	0.2	0.1	4.4
Agriculture+Business on one’s own	1.4	2.9	1.0	0.5	1.3	0.0	0.2	1.0
Agriculture+Fishery	0.0	0.0	0.0	4.7	2.2	0.0	0.0	1.2
Other Combination	2.1	3.2	1.6	5.9	2.4	1.1	1.1	2.6

**Table 19b.** Occupation of participants in cohort II females (%).

	Kasama	Kashiwa zaki	Tosa yamada	Arikawa	Miyako	Suita 1	Suita 2	Total
N	9,527	1,623	3,968	6,187	5,235	3,576	2,387	32,503
Agriculture	11.2	17.9	23.9	13.0	21.8	0.0	0.0	13.1
Forestry	0.1	0.1	0.1	0.1	0.1	0.0	0.0	0.1
Fishery	0.0	0.0	0.0	1.3	0.1	0.0	0.0	0.3
Employed	22.7	20.5	24.4	11.3	14.3	20.5	18.5	18.7
Business on one’s own	9.6	4.6	8.4	8.5	12.0	5.4	8.3	8.8
Expert	2.7	1.2	3.4	1.1	3.7	3.6	2.1	2.6
Housewife	30.7	18.1	23.1	37.4	27.1	53.8	55.3	34.2
Unemployed	7.6	5.1	3.7	6.8	4.6	1.0	7.2	5.6
Others	0.4	0.2	0.5	0.2	0.7	0.3	0.2	0.4
Agriculture+Employed	1.8	4.0	0.8	1.1	0.3	0.0	0.0	1.1
Agriculture+Business on one’s own	0.4	0.8	0.3	0.4	0.2	0.0	0.1	0.3
Agriculture+Fishery	0.0	0.0	0.1	0.6	0.0	0.0	0.0	0.1
Other Combination	12.9	27.6	11.4	18.2	14.9	15.4	8.4	14.7

Fruit and horticulture occupations were common in Tosayamada, and other types of agriculture were common in Miyako. Rice agriculture was a major occupation in other districts. In Suita subcohorts 1 and 2, the proportion of managers was high for males and that of desk workers was high in females, whereas the proportion of manual workers was high in other districts. In females, the proportion of housewives or unemployed persons was high, especially in urban districts such as Katsushika and Suita.

#### 3) Family Living in the Same House

In cohort I, the proportion of participants living with their parents was lower in Ishikawa and Katsushika than in other districts. In cohort II, the proportion of families in which husband and wife, children, and parents lived together was higher in Kashiwazaki, and the proportion of nuclear families with only husband and wife was higher in Arikawa. Suita subcohort 1 showed a high proportion of families in which husband and wife lived with children.

#### 4) Numbers of Siblings and Numbers of Children

In cohort I, the number of brothers and sisters and the number of children was highest in Ishikawa and lowest in Katsushika (Tables 20a,b and 21a,b).

**Table 20a.** Numbers of brothers and sisters of participants in cohort I males (%).

	Ninohe	Yokote	Saku	Ishikawa	Katsushika	Total
N	4,215	5,469	5,366	5,151	2,759	22,960
0-4	50.0	54.9	57.4	43.6	70.0	53.9
5-6	26.8	24.7	26.4	27.4	17.9	25.3
7-8	16.0	14.0	11.8	18.1	8.7	14.1
9-	7.3	6.4	4.3	10.9	3.5	6.7

Mean	4.7	4.5	4.2	5.0	3.6	4.5
S.D.	2.6	2.5	2.4	2.8	2.3	2.6

**Table 20b.** Numbers of brothers and sisters of participants in cohort I females (%).

	Ninohe	Yokote	Saku	Ishikawa	Katsushika	Total
N	4,865	6,282	5,446	5,291	3,892	25,866
0-4	49.1	53.0	56.4	44.2	71.8	54.1
5-6	27.6	25.8	27.3	26.5	18.7	25.5
7-8	15.3	14.7	12.0	18.6	7.5	13.9
9-	8.0	6.6	4.2	10.8	2.0	6.5

Mean	4.7	4.6	4.2	5.0	3.5	4.4
S.D.	2.7	2.5	2.3	2.8	2.2	2.6

**Table 21a.** Number of children of participants in cohort I males (%).

	Ninohe	Yokote	Saku	Ishikawa	Katsushika	Total
N	4,215	5,469	5,395	5,151	2,758	22,988
0	8.0	6.2	8.7	13.3	22.1	10.6
1-2	49.8	69.2	47.1	21.7	58.3	48.5
3-4	38.8	24.0	43.0	48.3	19.0	36.0
5-6	2.9	0.4	0.8	13.9	0.4	4.0
7-	0.5	0.1	0.4	2.9	0.3	0.9

Mean	2.4	2.1	2.3	3.0	1.7	2.3
S.D.	1.2	0.9	1.1	1.8	1.1	1.3

**Table 21b.** Number of children of participants in cohort I females (%).

	Ninohe	Yokote	Saku	Ishikawa	Katsushika	Total
N	4,865	6,282	5,468	5,291	3,982	25,888
0	6.2	6.3	5.7	8.6	9.5	7.1
1-2	48.2	65.2	48.0	22.1	63.6	49.3
3-4	41.2	27.5	45.0	47.7	26.2	37.7
5-6	3.8	0.8	1.2	17.5	0.5	4.8
7-	0.6	0.2	0.1	4.1	0.1	1.0

Mean	2.5	2.1	2.4	3.3	2.0	2.5
S.D.	1.2	0.9	1.0	1.8	1.0	1.3

#### 5) Education

In cohort I, the proportion of junior high school graduates was highest in Ninohe (64%). In Katsushika, the proportion of high school graduates was 41% and of university graduates was 19%. Katushika had a higher proportion of well-educated people than other districts. Education history was not included for cohort II, but Suita would be the same as other urban areas.

## DISCUSSION

Prevalence of hypertension, stroke, and cancer in the past and family history was relatively high in every district of cohorts I and II, whereas that of coronary heart disease (CHD) was low. A past history of hypertension and stroke was high in the Tohoku area and low in Okinawa, compared to the other districts. It coincided with the mortality data in these areas. The prevalence of past history of stomach and liver cancer was higher in Arikawa than other districts. Many participants in Arikawa work in fishery occupations, so further follow-up may find interesting risk factors, such as intake of burned fish.

The frequency of participants who received medication from doctors ranged from 20% to 30%, with higher rates in Tohoku areas and lower rates in Okinawa compared to the other districts. The most commonly used medicine was an antihypertensive drug. It accounted for about 50% of all medications. The prevalence of medication usage for high serum lipids and for diabetes mellitus was still low (around 10%). Participation in basic health examinations by the local governments showed a rate of over 70% in all districts. The rate was particularly high in the Tohoku area, where a high prevalence of a stroke had been present. It appears to be a result of long-term public health service through the public health center.

Despite the different questions about health examination in cohorts I and II questionnaires, the participation rate for examining blood pressure, blood chemistry, and chest x-ray was over 70% in all districts of cohort I. The participation rate for electrocardiographic and funduscopic examinations was higher in the Tohoku area than in the other areas. Participation rates for Barium examination of stomach, gastrointestinal endoscopy, occult blood examination in stools, barium examination of the colon, and colon endoscopy, and so forth, were high in Saku, where a comprehensive mass screening program for detecting early stomach and colon cancer has been extensively performed.

The participation rate for blood pressure measurement was high (over 85%) in both males and females in cohort II. The participation rate for serum cholesterol measurement was relatively high in Suita subcohort 2 (70.9% in males and 63.5% in females), and it was about 50% in other districts. The lowest was Arikawa (35%).

In every district of both cohorts I and II, the proportion of persons who recognized their own values of systolic and diastolic blood pressure was high, whereas a few persons remembered their own serum cholesterol levels. Explanation of the results by health professionals to participants was usually insufficient for serum cholesterol^11^^)^. Recognition of health examination results should enhance the prevention of CHD in Japan where serum cholesterol level had been rising in recent years^12^^)^.

The frequency of persons who participate in sports or physical exercise was high in Okinawa and Suita subcohort 2. However, the mean total physical activity (both at work and for leisure time) was lowest in Suita subcohort 2. It was suggested that participants in Suita subcohort 2 tried to do more sports and physical exercise because of their low physical activity at work.

The frequency of more active and positive persons was higher in urban districts and lower in Okinawa compared to the other districts. The association between personality and health status will be examined in a follow-up study.

In urban districts of Katsushika and Suita where there were many employed persons, the prevalence of hyperlipidemia and myocardial infarction in the past history was relatively high compared to the rural districts. On the other hand, the prevalence of hypertension and stroke in the past history was high in rural districts, especially in Tohoku areas such as Ninohe and Yokote where agriculture was dominant.

The results of the questionnaire investigation corresponded generally to the geographic characteristics and mortality profiles described in the third chapter of this book.

The association between the differences of health status, life habits, and social background and occurrence of various lifestyle-related diseases shall be clarified by a follow-up study in the JPHC study.
